# Targeting the DNA Damage Response for Cancer Therapy by Inhibiting the Kinase Wee1

**DOI:** 10.3389/fonc.2022.828684

**Published:** 2022-02-17

**Authors:** Amirali B. Bukhari, Gordon K. Chan, Armin M. Gamper

**Affiliations:** Department of Oncology, Cross Cancer Institute, University of Alberta, Edmonton, AB, Canada

**Keywords:** kinase, DNA damage response (DDR), cell cycle, cancer therapy, Wee1, synthetic lethality

## Abstract

Cancer cells typically heavily rely on the G2/M checkpoint to survive endogenous and exogenous DNA damage, such as genotoxic stress due to genome instability or radiation and chemotherapy. The key regulator of the G2/M checkpoint, the cyclin-dependent kinase 1 (CDK1), is tightly controlled, including by its phosphorylation state. This posttranslational modification, which is determined by the opposing activities of the phosphatase cdc25 and the kinase Wee1, allows for a more rapid response to cellular stress than *via* the synthesis or degradation of modulatory interacting proteins, such as p21 or cyclin B. Reducing Wee1 activity results in ectopic activation of CDK1 activity and drives premature entry into mitosis with unrepaired or under-replicated DNA and causing mitotic catastrophe. Here, we review efforts to use small molecule inhibitors of Wee1 for therapeutic purposes, including strategies to combine Wee1 inhibition with genotoxic agents, such as radiation therapy or drugs inducing replication stress, or inhibitors of pathways that show synthetic lethality with Wee1. Furthermore, it become increasingly clear that Wee1 inhibition can also modulate therapeutic immune responses. We will discuss the mechanisms underlying combination treatments identifying both cell intrinsic and systemic anti-tumor activities.

## Wee1, the Cell Cycle, and the DNA Damage Response

The cellular genome is exposed to insults by several endogenous (reactive oxygen species, DNA replication errors) as well as exogenous (chemical mutagens, ionizing radiation, ultraviolet light) DNA damaging factors. Ionizing radiation from cosmic radiations or medical treatments (X-ray scans or radiation therapy) can generate base lesions as well as single and double-strand DNA breaks. Additionally, cancer chemotherapeutics can intentionally induce a variety of DNA lesions, including inter- and intra-strand cross-links arising from drugs like cisplatin or Mitomycin C. To ensure safe passage of the genomic material to the next generation, all organisms have evolved mechanisms – collectively termed the DNA damage response (DDR) – to detect DNA damage and to activate a signaling cascade to promote repair, including *via* cell cycle checkpoint activation (1), or in the case of extensive DNA damage to trigger mechanisms to either permanently exit the cell cycle (senescence) or undergo programmed cell death (apoptosis), presumably preventing cells from accumulating mutations and resulting in the development of cancer.

The DNA damage response and the cell cycle are intimately linked through cell cycle checkpoints, “control mechanisms enforcing dependency in the cell cycle” (2). Of the four cell cycle checkpoints, only the spindle checkpoint in mitosis is not clearly linked to pathways activated by DNA damage. As most cells in a human are in G1 (G0) phase, the G1/S checkpoint will prevent most normal cells to enter the cell cycle after DNA damage. The pathways initiated by the apical kinases Ataxia Telangiectasia-mutated (ATM) (3, 4) and Ataxia telangiectasia and Rad3 related (ATR) (5) relay the damage signal to downstream effectors, including the tumor suppressor p53, a central node in the DNA damage response (6). These two kinases also play an important role in the S phase checkpoint (7–9), which ensuring accurate replication, and for the G2/M checkpoint (7, 10). The latter checkpoint prevents cells with damaged or under-replicated DNA to enter mitosis, an event which poses a high risk of chromosome aberrations (11). As all checkpoints are governed by cyclin-dependent kinases (CDKs), all DNA damage pathways ultimately converge on the regulation of the CDK activity. A dysregulated cell cycle is able to lead to DNA damage and genomic instability is a hallmark of cancer (12).

## The Kinase Wee1, a Gatekeeper at Several Cell Cycle Checkpoints

**Figure 1:**
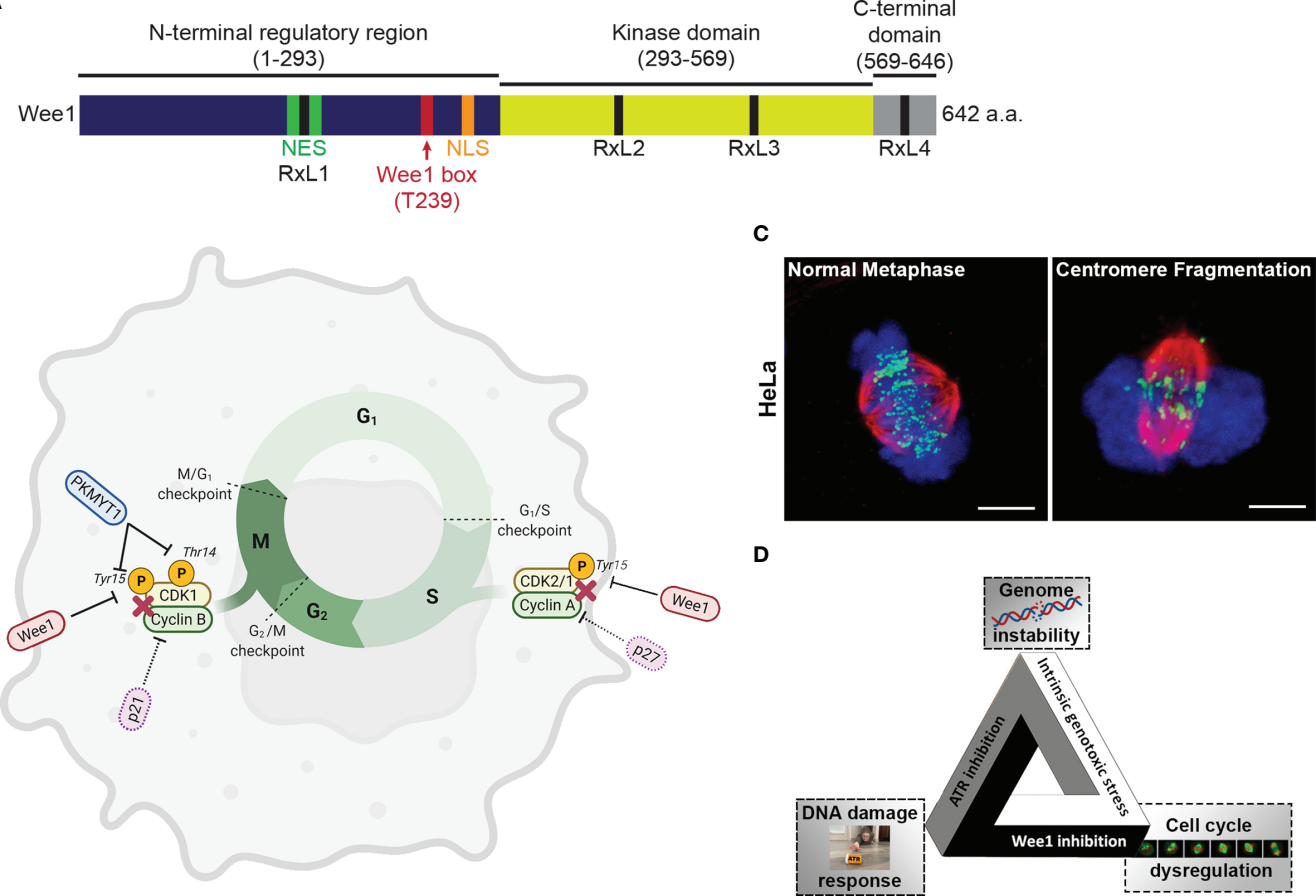
**(A)** The 642 amino acid long protein kinase Wee1 contains a N-terminal regulatory domain (dark blue), a kinase domain (yellow green), and a short C-terminal domain (gray). The diagram also shows a nuclear localization sequence (NLS; orange), a nuclear export sequence (NES; green), a highly conserved regulatory Wee1 box (red), and four cyclin binding motifs (RxL1, RxL2, RxL3, and RxL4; black). **(B)** Regulation of the cell cycle *via* phosphorylation of Cyclin-Dependent Kinases (CDKs) by Wee1 and the related protein kinase MYT1. **(C)** Images of HeLa cells in metaphase undergoing unperturbed mitosis or centromere fragmentation due to mitotic catastrophe as a result of premature entry into mitosis. (chromosomes, blue; tubulin, red; centromeres, green) **(D)** The fateful triangle underlying the conditional synthetic lethality observed in cancer cells leading to selective killing by combined ATR and Wee1 inhibition.

Wee1 is a tyrosine kinase originally discovered in *Schizosaccharomyces pombe* (13). Human Wee1 was subsequently discovered as a crucial regulator of the G2/M checkpoint (14). The primary structure of Wee1 is composed of an amino-terminal regulatory domain, a kinase domain, and a short C-terminal domain. The N-terminal domain coordinates signals to shuttle Wee1 into and out of the nucleus (15, 16). Wee1 contains four cyclin binding motifs, RxL1, RxL2, RxL3, and RxL4, to facilitate interaction with CDK (15) (**Figure 1A**).

The Wee kinase family comprises three serine/threonine kinases: *Wee1*, *PKMYT1*, and *Wee2*. In mammalian cells, Wee1 and PKMYT1 (protein kinase membrane-associated tyrosine/threonine 1; also known as Myt1) have a vital role in regulating the G2/M transition (17) (**Figure 1B**). Wee2 (or Wee1B) is only expressed in germ cells, where it prevents premature restart of oocyte meiosis prior to ovulation and permits metaphase II exit at fertilization (18). PKMYT1 functions as an essential component of an organelle-based cell cycle checkpoint to prevent CDK1-induced premature fragmentation of Golgi and the endoplasmic reticulum during the G2 phase (19). PKMYT1 negatively regulates CDK1 activity by phosphorylation on both threonine 14 and tyrosine 15 (20, 21) - unlike Wee1, which only phosphorylates CDK1 on tyrosine 15 rendering CDK1 inactive (22, 23). In the absence of DNA damage, CDK1 is dephosphorylated by the Cell division cycle 25 (Cdc25c) phosphatase resulting in CDK1/cyclin B activation and initiation of mitotic events (24). In the unperturbed cell cycle Polo-like kinase 1 (PLK1) phosphorylates Wee1 at the G2/M transition, which targets Wee1 for degradation *via* the ubiquitin proteasome system (25). PLK1 also phosphorylates and activates the phosphatase cdc25 resulting in CDK1 activation (25, 26). Furthermore, Wee1 has a role in regulating replication dynamics during S phase. During S phase, initiation of replication results in the firing of many replication origins triggered by the action of DBF4-Dependent cdc7 kinase (DDK) and CDK2, the main S phase CDK (26, 27). Wee1 and cdc25 control CDK2 activity by regulating the phosphorylation status at tyrosine 15 (28). Importantly, Wee1 downregulation triggers a DNA damage response resulting in DNA replication stalling and reduced replication fork speed and causes cells to accumulate in S phase (29). It was proposed that in unperturbed cells, Wee1 protects replication forks and prevents generation of DNA damage by inhibiting the Mus81 endonuclease (29). Several studies have indicated that Wee1 levels are regulated by non-coding RNAs, which could impact cellular sensitivity to genotoxic agents (30).

Cancers often have a deregulated G1 checkpoint. As a result, they are heavily reliant on the G2/M checkpoint for survival and mitosis. Consequently, Wee1 is often highly expressed in many cancers including breast (31, 32) and lung (31) cancers, glioma (33), melanoma (34), leukemia (35, 36), osteosarcoma (37), and squamous cell carcinoma (38). As most cancer therapies aim to induce lethal amounts of DNA damage in cancer cells, Wee1 overexpression promotes cancer cell survival (and resistance) by reinforcing DNA damage checkpoints and preventing mitotic catastrophe (33). The key role of Wee1 in regulating the G2/M checkpoint in response to DNA damage has made it an attractive target for cancer therapy. Despite its appeal, to date only one selective and highly potent small molecule Wee1 inhibitor, AZD1775 (also known as Adavosertib or MK-1775) (39), has been widely reported and is being evaluated against various advanced cancers in phase I/II clinical trials either as a monotherapy (40–42) or in combination with other chemotherapies (40, 43, 44). Yet several new inhibitors are being developed or are already making it into the clinic (see below).

## Wee1 Inhibition and Mitotic Catastrophe

Mitotic catastrophe is a major mode of tumor cell death following genotoxic treatments including irradiation (45). Mitotic catastrophe is loosely defined as cell death that occurs during or following an aberrant mitosis (46, 47). While its molecular mechanism is unclear, increasing evidence points to the involvement of caspases (46, 48). Wee1 knockout and the loss of Cdk1 T14 and Y15 phosphorylation causes ectopic Cdk1 activity, uncontrolled mitotic entry and cell death (14, 49–51). Similarly, Wee1 inhibition with AZD1775 or siRNA-mediated knockdown of Wee1 results in premature mitotic entry, prolonged mitotic arrest and mitotic catastrophe (33, 52–56). Furthermore, ectopic activation of Cdk1 and activation of the Mus81 endonuclease complex in S phase results in stalled DNA replication forks and DNA damage (29, 57). The ectopic Cdk1 activity induces replication stress and fork collapse through the depletion of dNTPs and aberrant replication origin firing (58–60), as Cdk1 phosphorylation of the ribonucleotide reductase subunit RRM2 induces its ubiquitin mediated degradation during DNA synthesis resulting in a 70% drop in dNTPs (60). Since Cdk1 activity induces chromosome condensation, ectopic Cdk1 activity also promotes premature chromosome condensation (61–63), generates torsional strain to the DNA backbone and results in DNA breakage (64, 65). Centromeres are late replicating due to a lower prevalence of replication origins and are prone to breakage during premature condensation or cleavage by the Mus81 endonuclease complex (64, 66). In a process known as checkpoint adaptation, cells with damaged DNA eventually escape the S and G2 checkpoint and enter mitosis prematurely (67). Checkpoint adaptation in both lower eukaryotes and mammalian cells has been consistently linked to the Plk1 [reviewed in (68)], the kinase phosphorylating Wee1 and promotes its ubiquitin mediated degradation through the SCF^βTrCP^ pathway (69, 70). Underlining the importance of this coordinated timing of kinases, Wee1 inhibition and subsequent premature entry into mitosis in the presence of under-replicated chromosomes can result in centromere fragmentation, a morphological marker of mitotic catastrophe (53, 71) (**Figure 1C**). Conversely, Wee1 overexpression can promote cell survival by reinforcing the DNA damage checkpoints and preventing mitotic catastrophe (33).

Wee1 inhibition by AZD1775 has been shown to induce *in vitro* and *in vivo* synergistic tumor cell killing with several DNA damaging therapies including IR (55) and chemotherapeutics like cisplatin, paclitaxel doxorubicin, 5-fluorouracil, and gemcitabine (53, 72–74). Given the role of p53 in regulating the G1 cell cycle checkpoint, treatment with AZD1775 has been reported to selectively target cancers harboring p53 mutations or loss of gene function (39, 75). Having said that, a few studies have also shown that AZD1775 sensitizes cancer cells to DNA damaging therapies independent of p53 status (76–78). Additionally, DNA damaging agents that specifically interfere with DNA synthesis and arrest cells in S-phase show high synergy with AZD1775 (59, 72). Overall, these preclinical studies support that AZD1775 has antitumor effects in a wide range of tumors both as a monotherapy and in combination with other chemotherapeutics.

## Wee1 Inhibitors in the Clinic

There are 60 clinical trials listed on clinicaltrials.gov (accessed January 2022) for AZD1775 where it is being evaluated against a wide range of cancer types including breast cancer, cervical cancer, leukemia, lung cancer, ovarian cancer, pancreatic cancer, pediatric and adult brain tumors (For a list of completed and active clinical trials with Wee1 inhibitors, see **Table 1**). Findings of phase I clinical trials show that AZD1775 is relatively well tolerated with acceptable toxicity profiles both as a single agent and in combination with other therapies (42). As a monotherapy, the maximum tolerated dose was determined as 225 mg, which was administered orally to ovarian cancer patients in five doses (2 twice per day, 1 once a day) per week over 3 weeks (42). The dose limiting toxicities included hematologic events, nausea, vomiting, and fatigue (40, 42). Interestingly, two of nine patients harboring *BRCA1* mutation recorded partial response, but unexpectedly none of the patients with documented p53 mutation exhibited a response (42). Early indications from a phase II trial evaluating AZD1775 plus carboplatin in p53 mutant ovarian cancer refractory or therapy-resistant patients show encouraging antitumor activity with one (5%) complete response and eight (38%) partial responses (43). Moreover, the overall response rate (43%) far exceeded the results that could be expected with second-line single agent treatments (11% to 21%) (43). A recent clinical trial evaluated the efficacy of AZD1775 as a monotherapy given once-daily as 5 days on and 2 days off in a 21 day cycle to patients (n = 42) with advanced solid tumors (79). The recommended phase II dose was determined as 300 mg, with most common toxicities including gastrointestinal and hematologic adverse effects. The dose-limiting toxicities included grade 4 hematologic toxicity and grade 3 fatigue (79). Six patients (14%; four ovarian and two endometrial cancers) confirmed partial response as the best response. Interestingly, one patient who progressed rapidly was found to have a Wee1 tumor mutation and potential compensatory PKMYT1 overexpression (79) (see below).

**Table 1:** Wee1 inhibitors in clinical trials.

Study Identifier	Co-Treatment	Tumor Type	Phase	Status
***Adavosertib (AZD1775) as Monotherapy***				
**NCT01748825**	–	S	1	Complete
**NCT03313557**	–	S	1	Complete
**NCT03668340**	–	S	2	Active
**NCT03315091**	–	S	1	Complete
**NCT02659241**	–	S	1	Active
**NCT03333824**	–	S	1	Active
**NCT02207010**	–	S	0	Complete
**NCT04439227**	–	S/H	2	Active
**NCT02593019**	–	S	2	Complete
**NCT04590248**	–	S	2	Active
**NCT03385655**	–	S	2	Active
***Adavosertib in combination with other cytotoxic therapies***				
**NCT02666950**	Cytarabine	H	2	Complete
**NCT01164995**	Carboplatin	S	2	Active
**NCT03012477**	Cisplatin	S	2	Complete
**NCT02101775**	Gemcitabine	S	2	Active
**NCT02906059**	Irinotecan	S	1	Complete
**NCT03330847**	Olaparib	S	2	Active
**NCT02341456**	Carboplatin/Paclitaxel	S	1b	Complete
**NCT02508246**	Cisplatin/Docetaxel	S	1	Complete
**NCT02194829**	Gemcitabine/Paclitaxel	S	1, 2	Active
**NCT03345784**	Cisplatin/RT	S	1	Active
**NCT02585973**	Cisplatin/RT	S	1	Complete
**NCT03028766**	Cisplatin/RT	S	1	Complete
**NCT02037230**	Gemcitabine/RT	S	1, 2	Complete
**NCT01849146**	Temozolomide/RT	S	1	Active
***Other Wee1 inhibitors in clinical testing – Zn-c3***				
**NCT04158336**	–	S	1	Active
***Other Wee1 inhibitors in clinical testing – IMP7068***				
**NCT04768868**	–	S	1	Active
***Other Wee1 inhibitors in clinical testing – Debio 0123***				
**NCT03968653**	Carboplatin	S	1	Active

Suspended trials were not included in the list. S, solid tumors. H, hematological cancers.

While AZD1775 is the most promising Wee1 inhibitor undergoing phase II clinical testing to date, its toxicity profile limits its use to intermittent dosing, potentially impacting clinical efficacy. Recently, Zentalis Pharmaceuticals reported the development of ZN-c3, a selective small molecule orally active bioavailable inhibitor of Wee1. Compared to AZD1775, which at higher concentrations also inhibits PLK1, a negative regulator of Wee1, ZN-c3 showed much higher selectivity for Wee1 over other kinases (80). Moreover, ZN-c3 demonstrated similar efficacy to AZD1775 *in vivo*. The expected superior safety profiles of ZN-c3 has enabled its quick transition to phase I/II clinical testing either as a monotherapy or in combination with other chemotherapies (NCT04158336). Similarly, a Wee1 inhibitor developed by Debiopharm, Debio 0123, is being tested in a phase I study (NCT03968653).

Early results for ZN-c3 of the phase I dose-escalation (25 mg to 450 mg) trial (NCT04158336) reported 300 mg as the recommended phase II dose (81). Of the 16 patients with post-baseline tumor assessments, 5 patients showed indications of stable disease, and 2 patients showed partial response. Interestingly, both the partial response patients had stage IV metastatic disease (colorectal cancer and ovarian cancer, respectively) and had undergone several lines of therapy (81). Importantly, ZN-c3 was reported to have higher selectivity and better safety profiles compared to AZD1775, making it particularly well suited for combination therapies (82). Out of the 39 patients involved in the trial, 30 experienced mild to moderate symptoms like nausea, diarrhea, vomiting, and fatigue (81). In addition to this, ZN-c3 is also undergoing clinical testing in combination with other chemotherapeutics like carboplatin, doxorubicin, paclitaxel, and gemcitabine in patients with platinum-resistant ovarian cancer (NCT04516447).

Other Wee1 inhibitors in clinical trials are Debio 0123 by the French company Debiopharm and, the most recent addition, IMP7068, developed by Impact Therapeutiucs in China. Schrödinger and Nuvation Bio have preclinical compounds that might soon advance soon in the pipeline as well (SDGR2 and NUV-569, respectively).

## Radiosensitization by Wee1 Inhibition

The G2/M checkpoint constitutes an important safeguard for preventing cells with damaged or under-replicated DNA to enter mitosis, particularly in cancer cells which often have an abrogated G1 checkpoint due to aberrations in p53 signaling, caused e.g. by mutations in the p53 gene, viral proteins or MDM2 overexpression. It is therefore not surprising that the first described Wee1 inhibitor, (non-selective) PD0166285, was tested in combination with ionizing radiation and was found to radiosensitize, i.e. to kill more efficiently, cancer cells in a p53-dependent manner (83). Studies with AZD1775 (39), which unlike PD0166285 does not inhibit the related kinase PKMYT1 as well, confirmed that inhibition of Wee1 leads to radiosensitization of a variety of cancer cells and increased radiation-induced tumor delay in mouse models (55). Since then several studies have shown that combining ionizing radiation with inhibition of Wee1 by AZD1775 increased cell death or clonogenic death of cells derived from a variety of cancers, including of the lung, breast, prostate, esophagus, cervix, liver, brain, and pancreas (55, 84–93). Yet it is not always clear in the mentioned studies whether the cooperativity was synergistic or just additive. (Only in the former case it would be appropriate to call the effect radiosensitizing.) Importantly, several preclinical studies in mice also showed increased tumor delay when radiation was combined with Wee1 inhibition (55, 85–87, 90–93) and several clinical trials are currently examining the efficacy of AZD1775 with radiation therapy (sometimes in combination with chemotherapy). Phase I trials produced promising results (94, 95), although the combination with cisplatin prompted the need for toxicity–related dosing adjustments (95). The initial proposal that p53 status was an essential biomarker for the radiosensitization by Wee1 inhibition (55) was put into doubt by subsequent studies (89). A likely explanation is that p53 status-independent defects of the G1 checkpoint could make cancer cells reliant on the G2/M checkpoint. Indeed, cyclin E overexpression renders cancer cells sensitive to Wee1 inhibition (96). Furthermore, several other mechanisms could lead to increased replication stress in cancer cells which would synergize with radiation and Wee1 inhibitor-mediated replication stress to endanger the survival of cancer cells entering mitosis. In this regard, the exact cellular mechanisms underlying the radiosensitization by Wee1 inhibition are still to be determined. For example, is the main reason for the cooperativity due to Wee1 inhibition lowering the G2/M transition threshold or increasing replication stress on top of ionizing radiation-induced DNA damage? Wee1 inhibition also suppresses homologous recombination (97), an important repair pathway particularly for radiation-induced double strand breaks. In the G2 phase homologous recombination could repair even complex DNA damage and DNA structures resulting from stalled or collapsed replication forks. The contribution of inhibition of these Wee1 roles, as well as known and yet to be identified crosstalk with other cellular pathways [e.g. autophagy (93)], to radiosensitization is likely specific to the cell type or even to the subpopulation (given tumor heterogenicity). Of importance for the clinic – and unfortunately much less characterized - is the heterogeneity in the radiosensitization of normal cells (between cell type and between persons) by inhibitors of cell cycle regulators. Of particular concern for normal tissue complications are potential deleterious effects of combining Wee1 inhibitors and radiation on stem cells, which often rely on an intricate crosstalk between external signaling factors and the cell cycle machinery to regulate their differentiation potential (98). As tissue homeostasis is usually dependent on tissue specific stem cells, the impact of Wee1 inhibition in the clinic must also be seen in the context of the heterogeneity in the radiation response within the stem cell compartment and plasticity (reviewed in (99)). Radiosensitizers are only useful for cancer therapy if they improve the therapeutic index in the current highly conformal treatment plans in radiation oncology.

## Synthetic Lethality With Wee1 Inhibition

Synthetic lethality refers to an interaction between two genes when the perturbation of either gene alone is viable but the simultaneous perturbation of both genes (gene functions) leads to cell death. A well-known example is deficiency of homologous recombination proteins BRCA1 or BRCA2 causing cancer cell sensitivity to poly (ADP-ribose) polymerase (PARP) inhibitors (100, 101). This distinctive synthetic lethality led to a strong interest in therapeutic approaches targeting cancer cells with other deficiencies in DNA damage response (DDR) pathways by inhibition of the alternative DDR pathway. Yet as this approach only targets cells with a specific defect in the DDR, unless loss of gene function leads to the cell-of-origin or occurs early in carcinogenesis, in which case the gene defect would be in all or most tumor cells, it is bound to only affect a subset of populations within a heterogeneous tumor. For example, the loss of Ataxia Telangiectasia-mutated (ATM) frequently observed in a variety of cancers (102) is likely due to a driver mutation occurring at an early stage during lung carcinogenesis (103). It therefore is expected that most cancer cells in those tumors will be killed by targeted drugs showing high efficacy in the background of defective ATM (104). Yet except for the ATM and p53 pathways (alterations in ATM, CHEK2, p53, MDM2), the majority of driver mutations in DNA damage response and repair genes were found to be subclonal in non-small cell lung cancer (103). It is to be expected that targeting an evolutionary late occurring gene defect, even if found in the subpopulation that constitutes the bulk of the cancer cells, would lead to the selection for the subpopulation with the functional gene and treatment resistance to follow. Furthermore, even in a homogenous population with a common DDR defect resistance can arise by reactivation of the defective pathway, as was observed in some PARP inhibitor resistant breast cancers (105). *Conditional* synthetic lethality refers to synthetic lethality observed only under certain circumstances, such as genetic background or metabolic state of cells or cellular environment (106). An approach to build synthetic lethality around cancer-intrinsic characteristics has the potential to decrease the probability for the tumors to acquire resistance. In that regard, one of the most common features of cancer cells is oncogene-induced DNA damage (107, 108), often leading to levels of replication and mitotic stress not observed in normal proliferating cells. This tumor-specific property makes it an ideal selective condition to base a synthetic lethality on to achieve a favorable therapeutic index.

An example of a successful attempt to establish a fateful triangle for cancer cells in a conditional synthetic lethality approach is the combination of inhibitors of Wee1 and of the kinase Ataxia telangiectasia and Rad3 related (ATR). This multi-pronged attack takes advantage of three features of cancer cells to selectively target them: genomic instability, dysregulated cell cycle and the reliance on particular DDR pathways for survival (**Figure 1D**). In a 2008 review discussing genomic instability, a designated hallmark of cancer (12), Halazonetis, Gourgoulis, and Bartek pointed out that, based on their data and the literature on observations in many cancer cell lines and precancerous and cancerous lesions from patients, “the presence of DNA damage was a feature that could distinguish precancerous lesions and cancers from normal tissues, irrespective of their proliferation rate” (108). DNA damage (genotoxic stress) is therefore a fundamental characteristic of cancer cells, unlike some other hallmarks of cancer which, due to tumor heterogeneity, may not manifest in every tumor or every tumor cell.

ATR is an apical kinase in the DDR and is activated by replication protein A (RPA)-coated single-stranded DNA, structures that can arise from stalled replication forks or resected DNA double-strand breaks (1). Not surprisingly, ATR plays a crucial role in the response to replication stress – likely the reason for it being an essential gene (109, 110). As a result, cancer cells rely on functional ATR signaling, particularly as other DNA damage response pathways are lost (such as the p53 and/or ATM pathway). This is exemplified by the importance of ATR signaling for the survival of cancer cells to ionizing radiation (5). Unsurprisingly, ATR activity is often upregulated in cancer cells (111, 112), including in cancer stem cells (113). ATR regulates Chk1 activity by phosphorylation of Chk1 kinase at serines 317 and 345 (1). Chk1 in turn targets Cdc25, the phosphatase removing inhibitory phospho-groups from cyclin-dependent kinases (CDKs), for degradation by phosphorylation-dependent ubiquitination. Because CDKs, particularly CDK1 and CDK2, regulate entry into mitosis and replication origin firing, Chk1 activation thereby prevents cell cycle progression (114). Thus, ATR/Chk1 signaling initiated at structures containing single-stranded DNA controls the S and G2 phase cell cycle checkpoints in mammalian cells (114). Importantly, the phosphorylation state of CDKs 1 and 2 (and thus their inhibition) is regulated by the balance between the kinase activity of Wee1 (and Myt1) and the phosphatase activity of Cdc25. The observed synergistic effects of Wee1 and ATR inhibition (71, 115) on cancer cell killing are surely in grant part due to the lowering of the threshold for CDK activation by combining inhibiting the constitutive phosphorylation and preventing checkpoint activation by the ATR/CHK1/Cdc25 axis, as combined AZD1775 and AZD6738 treatment leads to mitotic catastrophe in cancer cells (71). Yet both Wee1 and ATR regulate other cellular aspects that will play a role, including their activities during replication: For example, the above mentioned role of Wee1 during S phase, including replication fork protection, as well as reportedly in timing the entry into S phase (116) are perturbed by AZD1775 and lead to substantial replication stress. ATR on the other hand, besides the many functions during unperturbed replication (117), also regulates DNA damage repair by promoting extensive DNA end-resection needed for homologous recombination (5, 118, 119). By utilizing the reversibility of Wee1 and ATR inhibition, we characterized the contributions of inactivation of each kinase and during different phases of the cell cycle, thus studying how abrogation of ATR and Wee1 activity cooperatively leads to cell death caused by mitotic defects (71). The findings are compatible with a model, where synergistic killing by ATR and Wee1 inhibitors is based on an increase in the DNA damage level while simultaneously lowering the DNA damage response capacity leading to mitotic catastrophe. This is achieved by Wee1 inhibition-induced DNA damage during replication, abrogation of ATR-mediated S phase checkpoint activation, inhibition of ATR-dependent homologous recombination, and amplified by increased entry into mitosis with defective genomes due to combined inhibition of ATR and Wee1. As high replication stress in cancer cells - due to the high level of baseline DNA damage *per se*, but also to the resulting exhaustion of factors needed for both repair and replication, such as RPA (120) – contrasts from the stress in normal cells, even in highly proliferative tissues, and cancer cells often have an increased reliance on the G2/M checkpoint, a therapeutic window is achieved in an example of a conditional synthetic lethality.

Several studies have also investigated whether defects in specific pathways sensitize to Wee1 inhibition. A recent study in basal-like breast cancer cells suggests that loss of PTEN may be one of the strongest markers of Wee1 inhibitor sensitivity (121). This might not come as a surprise, given the role PTEN plays in replication progression and several studies showing that PTEN loss increases replication stress (122–124). A recent study also showed that HPV16 positivity sensitizes head and neck squamous cell carcinomas to Wee1 inhibition by a mechanism involving a circuit linking CDK1 and FOXM1 (125), a master transcriptional regulator of mitotic genes (126). Fitting with the scheme of vulnerability to Wee1 inhibition based on an already dysregulated cell cycle, an unbiased screen identified several S phase genes as determinants for AZD1775 sensitivity (127). Related, another screen identified defects in the Fanconi anemia pathway and in homologous recombination, mechanisms needed for effective DNA replication particularly in the background of increased DNA damage, as sensitizing to Wee1 inhibition (128). A strategy of releasing tumor cells from a cell cycle block into a phase where the cells are sensitive to Wee1 inhibition was used in a preclinical study with sarcoma. The combined sequential treatment with the CDK4/6 inhibitor Palbociclib and AZD1775 showed at least additive effects on tumor growth (129). In a model for the clinical scenario of breast cancer cells resistant to endocrine therapy and CDK4/6 inhibitors, derived long-term estrogen deprived endocrine resistant cell lines were found to be more resistant to CDK4/6 inhibitors, but more sensitive to AZD1775 or Wee1 knockdown than their parental cell lines (130). An interesting observed synergistic interaction was found between AZD1775 and A1155463 in cancer cells from a genetically engineered animal model for triple negative breast cancers (131). A1155463 is an inhibitor of the anti-apoptotic BCL-X_L_ protein (132). The drug combination also showed efficacy *in vivo*, but unfortunately the authors did not report the effect of the individual drugs in their mouse model (131). AZD1775 was also found to synergize with the PARP inhibitor olaparib in a xenograft model for triple negative breast cancer (133).

## Resistance Mechanisms to Wee1 Inhibition

Obvious candidate resistance mechanisms to Wee1 inhibitors include reversal of expression profiles of genes that are the base for Wee1 inhibitor vulnerability. For example, while cyclin E overexpression sensitizes cancer cells to AZD1775, reducing cyclin E levels has the opposite effect (96). A mechanism of *acquired* AZD1775 resistance observed both *in vitro* and *in vivo* is *via* the upregulation of PKMYT1 (53). As mentioned, Wee1 and the related kinase PKMYT1 exhibit functionally redundant roles in the inhibition of CDK1/cyclin B, the mitosis promoting complex (134–136). Yet compared to Wee1, PKMYT1 is much less studied in the context of cancer biology. This might be due to reports that inhibition or knockdown of Wee1 alone is sufficient to abrogate the S- and G2/M DNA damage checkpoints and that the loss of PKMYT1 neither affects the timing of mitosis nor abrogates DNA damage checkpoints in the presence of Wee1 (56, 137–139). On the other hand, combined knockdown of Wee1 and PKMYT1 causes more HeLa cells to enter mitosis with damaged DNA compared to Wee1 knockdown alone (56), PKMYT1 knockdown enhances AZD1775 induced cell killing in cell lines derived from brain metastases (140), and PKMYT1 is essential for cell survival in a subset of glioblastoma cells that have downregulated Wee1 expression (141). The protective mechanism by PKMYT1 upregulation leading to AZD1775 resistance was found to be due to compensatory inhibition of ectopic CDK1 activity by PKMYT1, allowing cells to escape mitotic catastrophe, the mode of cell death induced by Wee1 inhibition (53).

It was proposed that cancer stem cells, which often show increased chemo- and radiation resistance compared to bulk cancer cells and due to their cellular plasticity and tumor initiating capability can lead to tumor relapse (142), could be targeted by Wee1 inhibition (143). Only a few studies have examined the efficacy of Wee1 inhibition – alone or in combination - in the eradication of cancer stem cells. Early findings that Wee1 inhibition by the unspecific inhibitor PD0166285 radiosensitizes glioma stem cells (CD133 enriched glioma neurospheres) (33) were contradicted by a study using AZD1775 (and glioma cell lines enriched for neuronal stem cells) (92). In contrast, another study found that glioma stem cells (unlike neuronal progenitor cells) were sensitive to Wee1 inhibition alone (141). Our studies in breast cancer showed that breast cancer stem cells were less sensitive to AZD1775 compared to bulk cancer cells, which could be due to reduced drug uptake or decreased reliance on Wee1 signaling. Interestingly, combined Wee1 and ATR inhibition was as toxic to cancer stem cells as to bulk breast cancer cells, potentially explaining the antimetastatic effect of the combination treatment (71). To our knowledge, this was the first report of a higher drug synergy observed in cancer stem cells compared to bulk cancer cells, compensating for the reduced sensitivity of cancer stem cells to the individual drugs. A recent study found that trastuzumab resistant breast cancer cell lines were enriched in cancer stem cells, but on average showed greater sensitive to AZD1775. AZD1775 treatment disrupted stem like properties in the tested trastuzumab resistant breast cancer cell lines (144). These studies indicate that insights into the role of Wee1 in cancer stem cell maintenance and the associated correlation with drug resistance could have a significant impact in the clinic.

As the ongoing clinical trials will provide data and samples from patients treated with AZD1775 (Adavosertib), not only will predictive biomarkers be identified, but it will also become clearer which are the preferred pathways for resistance acquisition to single agent therapies. This in turn will provide important clues for improved treatment plans with combination therapies.

## Wee1 Inhibition - Beyond Cell-Intrinsic Cytotoxicity

The interaction between tumor cells and immune cells plays a determining role not only during carcinogenesis, where the survival of transformed cells is based on immune evasion, but also in cancer therapy, where the immune system is a key factor in achieving local and systemic tumor control. Several pathways involved in both DNA damage repair/signalling and immunity indicate that the immune system and the DNA damage response (DDR) have coevolved, resulting in processes with overlapping enzymatic networks. Examples range from prokaryotic defense systems, such as the antiviral CRISPR machinery, to the complex mammalian immune stimulation and maturation processes, such as class-switch recombination. A classic case in point is the discovery in 1995 that the lack of DNA-PK caused both extreme radiosensitivity and severe combined immunodeficiency (145, 146). Since then several cellular links between proteins in the DNA damage response and immune signaling have been uncovered. Of particular interest is the stimulator of interferon genes (STING) pathway, that can be activated by cyclic GMP-AMP synthase (cGAS) binding to DNA fragments and the subsequent production of the allosteric modulator of STING, the small messenger molecule cGAMP (147). This pathway was discovered as an important defense mechanism against DNA viruses, but was later found to get activated by DNA damage in the nucleus or mitochondria as well (148, 149). Besides cGAS, which binds to the DNA backbone, STING activation by DNA fragments might also involve the recognition of DNA ends by DNAPK (150) and/or the MRN complex (151). Kinases downstream of STING, TANK binding kinase 1 and IκB kinase, induce the transcription of genes involved in the innate immune response, such as interferons, interleukins and TNF, *via* the transcription factors IRF3 and NFκB (152). Several studies have shown that exogenous and endogenous genotoxic stress can induce the expression of interferon-stimulated genes, including stress due the loss of genes involved in the DNA damage response [reviewed in (153)]. Furthermore, activation of the apical DDR kinases ATM and ATR can also lead to upregulation of PD-L1 (*via* the STAT1 and STAT3 pathway) (154) and natural killer group 2D (NKG2D) ligands (155, 156).

Besides these DNA damage-induced changes in surviving cells, therapy-induced cell death itself can have a big immunomodulatory effect. The Nomenclature Committee on Cell Death defines immunogenic cell death (ICD) as “a functionally peculiar form of regulated cell death that is sufficient to activate an adaptive immune response specific for endogenous (cellular) or exogenous (viral) antigens expressed by dying cells” (45). Besides the release of antigenic determinants, such as neoepitopes, dying tumor cells also can lead to a local release of damage-associated molecular patterns and cytokines resulting in local effects on immune cell trafficking and activation. The observation that inhibition of Wee1 increases replication stress as well as the likelihood of untimely entry into mitosis, raising the possibility of DNA structures activating the STING pathway as well as mitotic catastrophe, make Wee1 inhibition a good candidate drug to increase the antitumor immune response. This makes Wee1 inhibition especially attractive to be combined with radiation therapy, as the latter is well known to be particularly inducive to ICD and Wee1 inhibitors are, as discussed previously, also radiosensitizing (39, 89, 157). Indeed preclinical studies have shown immune stimulating effects of Wee1 inhibition in combination with irradiation (158, 159). The exact mechanisms underlying the increased anti-tumor immunity, including the extent interferon signaling is involved, are still unclear. Of note, a recent study showed that inhibition of Wee1 alone failed to induce a type I interferon response, despite increasing DNA double strand breaks, cytosolic DNA, and micronuclei – all cellular phenotypes previously correlated with STING pathway activation (160).

## Conclusion

In conclusion, Wee1 inhibitors show great potential to make an impact in the clinic for the therapy of several cancer types. While some concerns have arisen from phase I/II clinical trials regarding potential side effects, it remains to be seen whether newer Wee1 inhibitors with supposedly higher kinase selectivity show an improved safety profile. Yet the most promising path are combination therapies allowing lower dosing of the Wee1 inhibitor than in monotherapy. Furthermore, optimization of the treatment plans, such as intermittent dosing of the Wee1 inhibitor, might improve the drug tolerance.

Regarding the kinase itself, still many questions remain to be elucidated on the biological role of Wee1, which revealed itself to be a multifaceted player during several phases of the cell cycle. Of special interest are the redundant and divergent roles of Wee1 and the related kinase PKMYT1, in normal tissues and in various cancer types.

## Author Contributions

AB, GC, and AG wrote and reviewed the manuscript together. All authors contributed to the article and approved the submitted version.

## Funding

ABB is supported by Alberta Cancer Foundation’s Dr. Cyril M. Kay Graduate Scholarship. GKC and AMG are funded by the Canadian Institute of Health Research and the Natural Sciences and Engineering Research Council of Canada, as well as the Cancer Research Society and Women and Children’s Health Research Institute (AMG).

## Conflict of Interest

The authors declare that the research was conducted in the absence of any commercial or financial relationships that could be construed as a potential conflict of interest.

## Publisher’s Note

All claims expressed in this article are solely those of the authors and do not necessarily represent those of their affiliated organizations, or those of the publisher, the editors and the reviewers. Any product that may be evaluated in this article, or claim that may be made by its manufacturer, is not guaranteed or endorsed by the publisher.
